# Supplementary figures and images for: A polymorphic residue that attenuates the antiviral potential of interferon lambda 4 in hominid lineages
Source: PLoS Pathog. 2018 Oct 11;14(10):e1007307. doi: 10.1371/journal.ppat.1007307 (PMC6181419; doi:10.1371/journal.ppat.1007307)

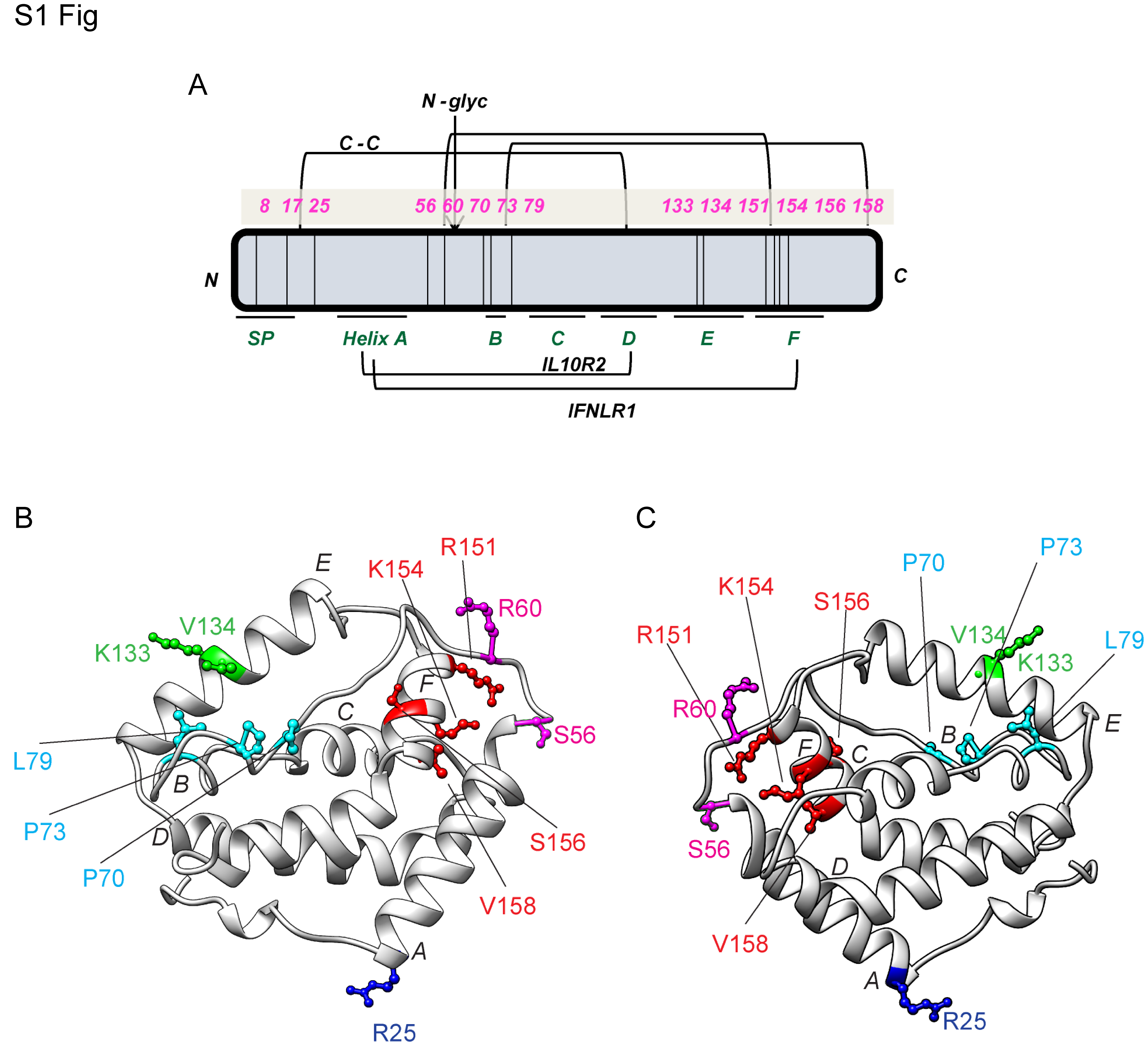

Supplement: S1 Fig — (A) Schematic location of non-synonymous variants in the HsIFNλ4 polypeptide (N- to C- terminus) (above schematic in pink). Regions of predicted structural significance are underlined, including the signal peptide (SP), single N-linked glycosylation site (N-glyc, arrowed), helices (A to F) and disulphide bonds (C-C). Note that there are 2 non-synonymous changes at C17 (C17R and C17Y). Helices involved in receptor interactions (IL10R2 and IFNλR1) are highlighted. (B and C) Location of non-synonymous variants on a homology model of HsIFNλ4 (side chains in colour) from two perspectives. Model was generated using the SWISS-MODEL online software. Helices are labelled A to F. Positions are coloured based on spatial clustering in the primary amino acid sequence. (TIF) [file ppat.1007307.s001.tif]

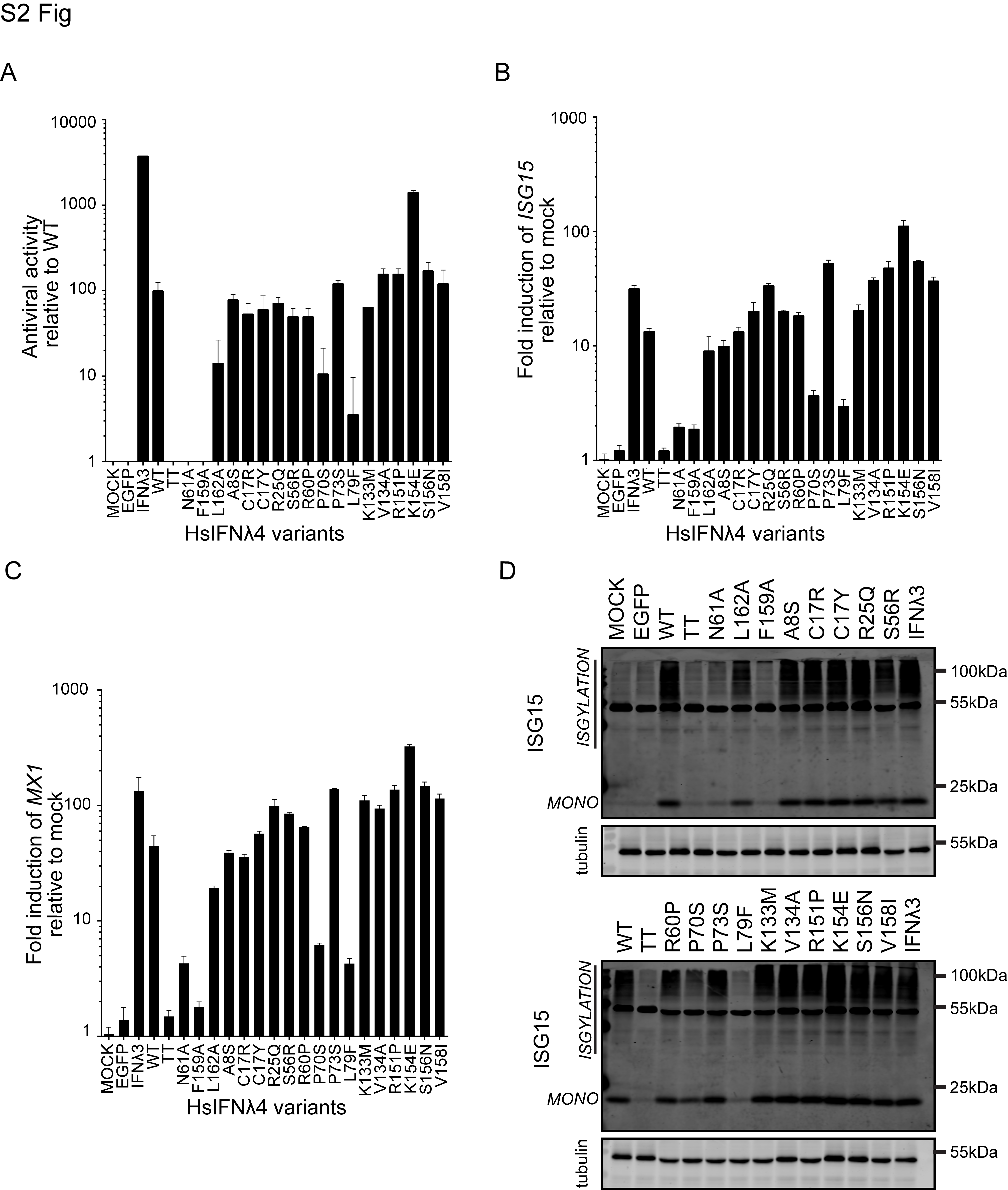

Supplement: S2 Fig — For data shown in panels A-D, all naturally-occurring variants of HsIFNλ4 were tested in antiviral and ISG induction assays. Experimental conditions included a series of controls including HsIFNλ3op (positive control), EGFP and the HsIFNλ4 TT variant (negative controls) as well as non-natural variants of HsIFNλ4 (N61A, F159A, L162A). N61A abrogates glycosylation of HsIFNλ4 while F159A and L162A are predicted to reduce interaction with the IFNλR1 receptor subunit and hence lower activity based on previous studies [27]. Panels show data from the following assays: (A) Antiviral activity in an anti-EMCV CPE assay in HepaRG cells. Cells were stimulated with serial dilutions of HsIFNλ4-containing CM for 24 hrs and then infected with EMCV (MOI = 0.3 PFU/cell) for 24 hrs at which point CPE was assessed by crystal violet staining. After staining, the dilution providing ~50% protection was determined. Data are shown as mean +/- SD of three independent experiments performed on different days. (B and C) ISG gene expression determined by RT-qPCR following stimulation of cells with HsIFNλ4 variants. Relative fold change of ISG15 mRNA (B) or Mx1 (C) in HepaRG cells stimulated with CM (1:4 dilution) from plasmid-transfected cells compared to wt HsIFNλ4. Cells were stimulated for 24 hrs. Error bar represent mean +/- SD of biological replicates (n = 3). (D) Western blot analysis of unconjugated and high molecular weight conjugated-forms of ISG15 (‘ISGylation’) from lysates harvested from HepaRG cells stimulated with CM (1:4) for 24 hrs. (TIF) [file ppat.1007307.s002.tif]

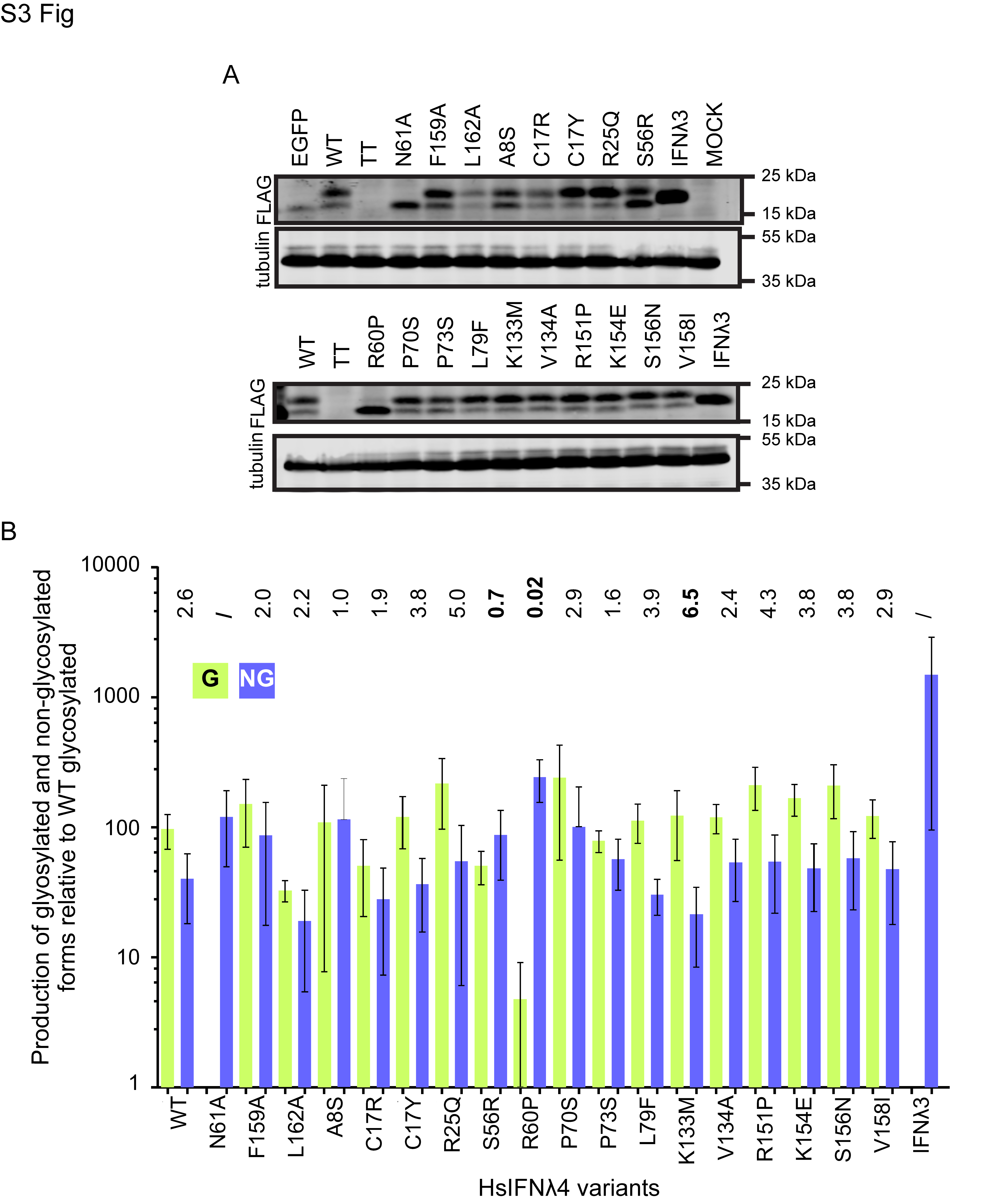

Supplement: S3 Fig — For data in panels A and B, expression and glycosylation of all naturally-occurring variants of HsIFNλ4 were examined. Experiments included a series of controls including HsIFNλ3op (contains no glycosylation sites), EGFP and the HsIFNλ4 TT variant (negative controls) as well as non-natural variants of HsIFNλ4 (N61A, F159A, L162A). N61A is predicted to abrogate glycosylation of HsIFNλ4. Panel A shows a representative Western blot for the production and glycosylation of HsIFNλ4 variants of lysates from plasmid-transfected producer HEK293T cells as detected with an anti-FLAG (‘FLAG’) primary antibody. Tubulin was used as a loading control. A non-specific band in the EGFP-transfected extract is shown (*). Panel B shows the quantification of intracellular glycosylated (green) and non-glycosylated (blue) HsIFNλ4 variants by Western blot analysis of lysates from plasmid-transfected producer HEK293T cells. Ratio of glycosylated to non-glycosylated is shown above the graph. Two- fold differences from wild-type are highlighted in bold. Data shown are mean +/- SEM combined from three independent experiments. (TIF) [file ppat.1007307.s003.tif]

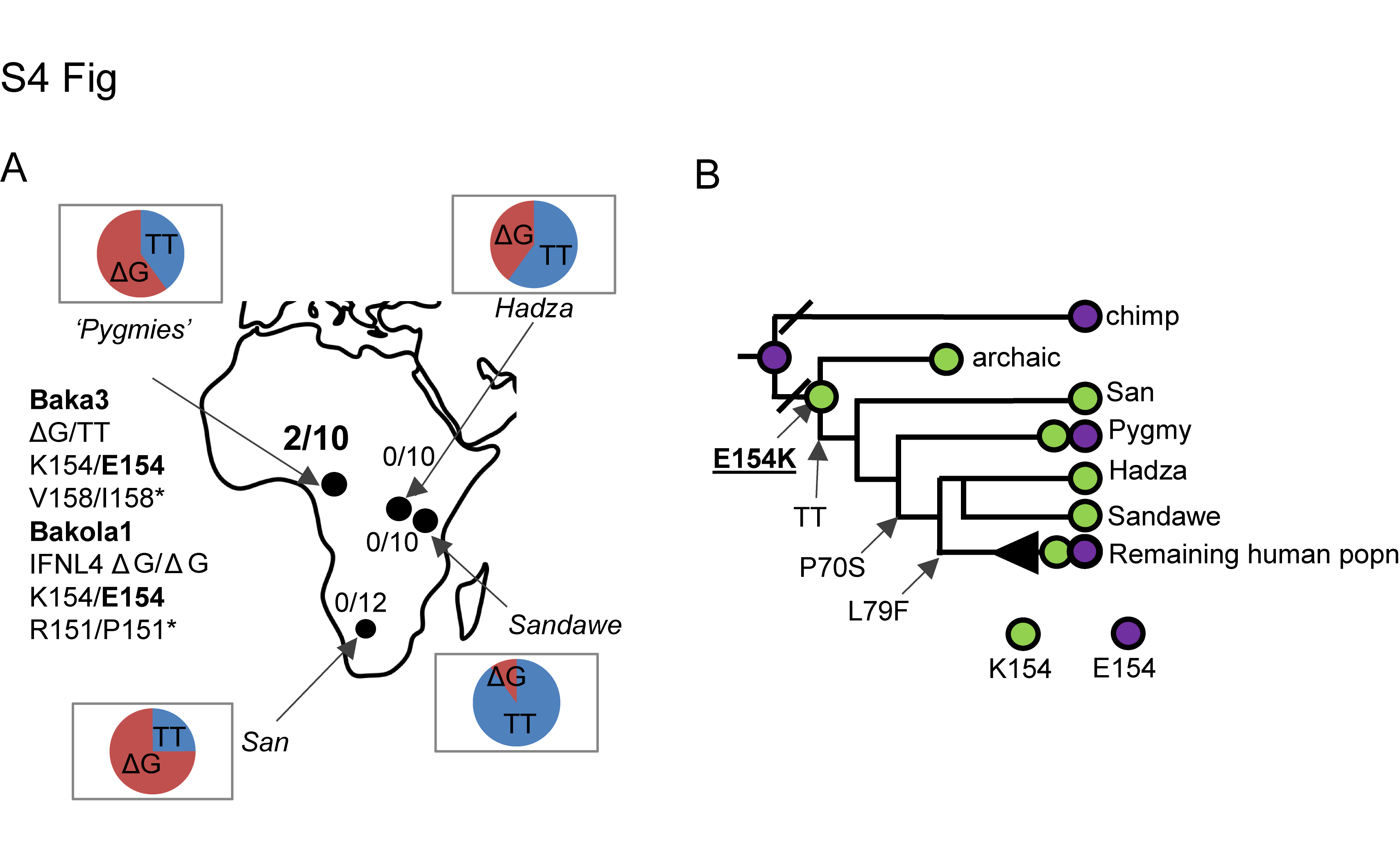

Supplement: S4 Fig — (A) Geographical location and frequency of HsIFNλ4 K154E in African hunter-gatherer alleles (Pygmy, n = 5 individuals, Sandawe (S) n = 5 individuals and Hadza (H) n = 5 individuals). Two Pygmy individuals within two tribes (Baka and Bakola) were found to encode the HsIFNλ4 K154E variant. The proportion of ΔG (red) and TT (blue) IFNL4 alleles are also shown in pie-charts. (B) Presence of HsIFNλ4 E154 (purple) versus HsIFNλ4 K154 (green) on a cladogram of human and chimpanzee evolution. Archaic human (Neanderthal and Denisovan) as well as other basal human populations (San, Sandawe and Hadza) only encode HsIFNλ4 K154. Earliest detection of the HsIFNλ4 TT frameshift and activity-reducing HsIFNλ4 P70S and HsIFNλ4 L79F variants are shown. All analysis can be found in S1 Data. (TIF) [file ppat.1007307.s004.tif]

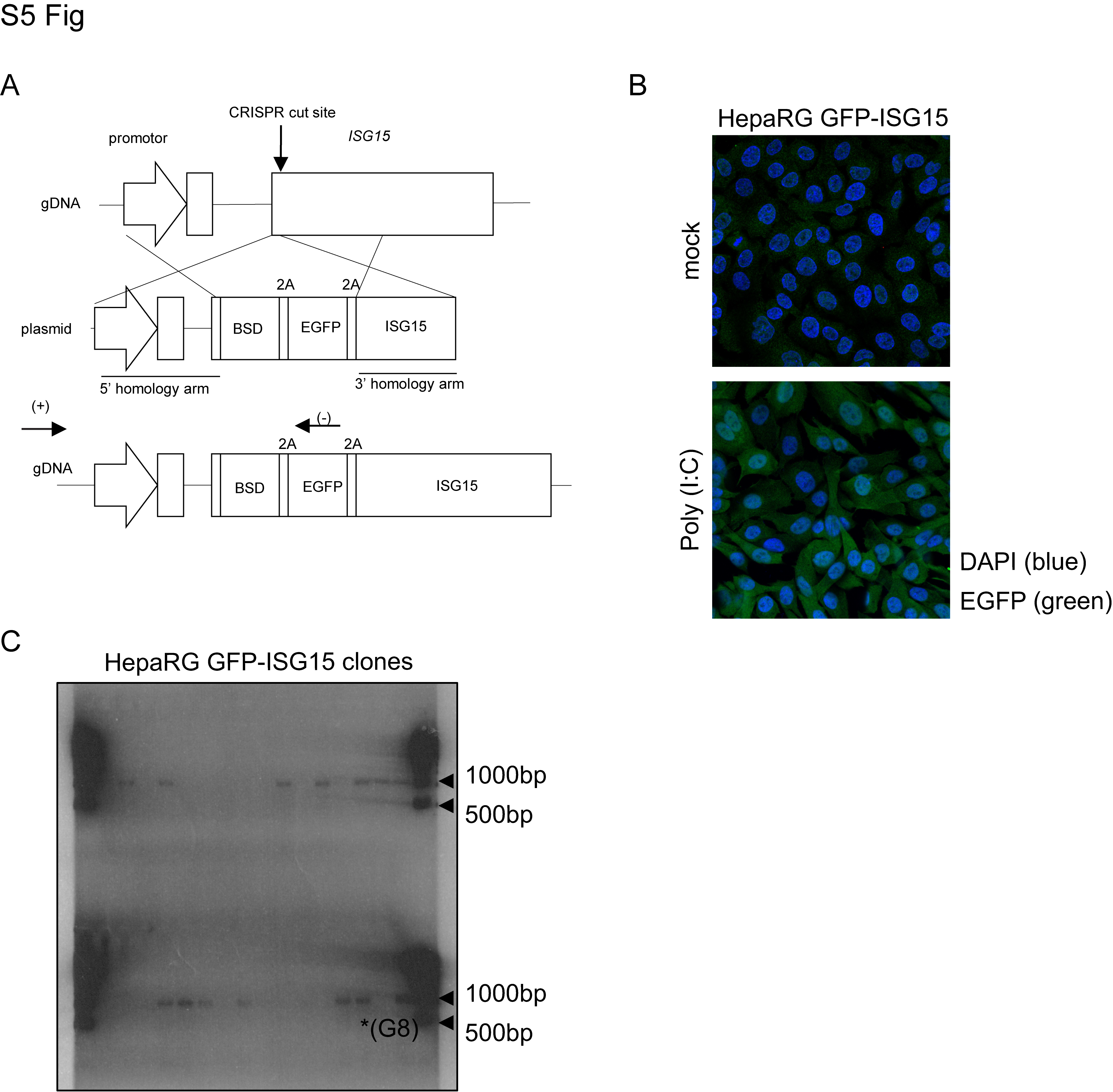

Supplement: S5 Fig — (A) Strategy for CRISPR-Cas9 genome editing combined with homologous recombination insertion of DNA sequences to produce an EGFP-expressing ISG15 promotor cell line. The strategy enables the insertion of a cassette in-frame with the ISG15 ORF that encodes blasticidin resistance (BSD) and EGFP genes followed by ISG15, separated by ‘2A’ ribosomal skipping sequences. Target gDNA and CRISPR-Cas9 cut sites are shown on the upper cartoon. Binding sites of primers for genotyping are highlighted (+) and (-) in the resulting modified gDNA. (B) Induction of EGFP expression (green immunofluorescence) in the G8 cell clone with or without treatment with PolyI:C (1.0 μg/ml for 24 hrs). Poly(I:C) stimulates the Toll-like receptor TLR3, which induces ISG15 expression. Cells were fixed in formalin, permeabilised and stained for indirect immunofluorescence with an anti-EGFP primary antibody before addition of a secondary antibody. DAPI was used as a counter stain for cell nuclei. (C) Cell line clone (G8) used for reporter assays was validated by PCR analysis yielding an amplified DNA fragment of ~1000 bp from primers located in ISG15 and EGFP sequences. (TIF) [file ppat.1007307.s005.tif]

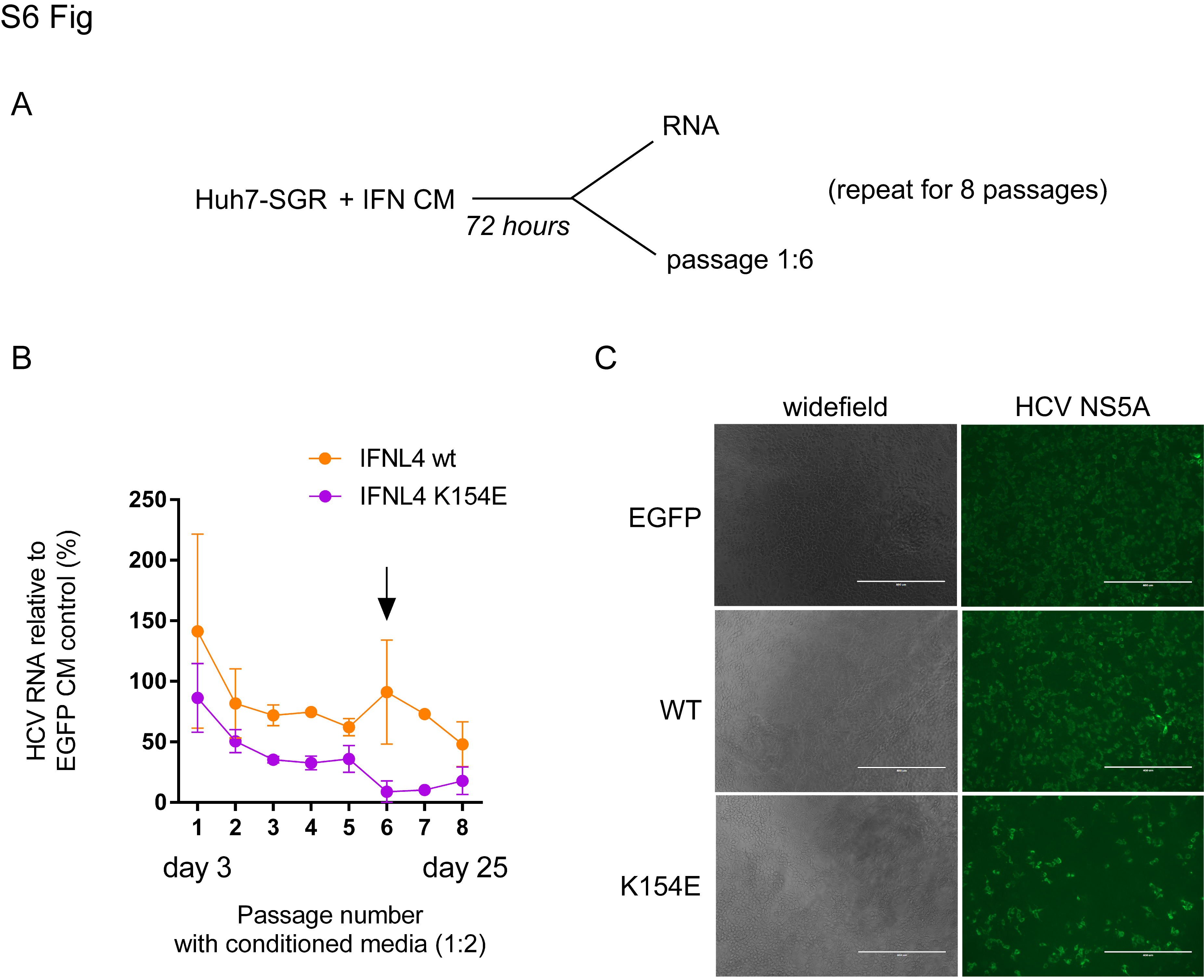

Supplement: S6 Fig — (A) A schematic of the experiment showing passaging of Tri-JFH1 Huh7 cells in the presence of HsIFNλ4 is shown. (B) Briefly, Tri-JFH1 cells were treated with CM containing wt HsIFNλ4 or HsIFNλ4 K154E alongside a negative control (EGFP) at a dilution of 1:2 for three days before repeat passaging at a ratio of 1:6 into EGFP- or HsIFNλ4-containing CM, again at a 1:2 dilution. This treatment and passaging were repeated 8 times over a total of 25 days. Cells were grown in 6 well plates. At each passage, a proportion of cells was stored for RNA extraction and quantification of HCV SGR RNA levels by RT-qPCR. HCV RNA relative to GAPDH was quantified for wt (orange) and K154E (purple) HsIFNλ4-treated cells, and expressed relative to the amount of RNA in the paired EGFP-treated cells. The arrow indicates a stage in the experiment where the cells were incubated for 4 instead of 3 days and reached confluency, which likely reduced HCV replication. The data show the mean and SD of biological duplicates. (C) At the end of the experiment (P8, 25 days), the cells were plated into a 96 well plate without either wt or K154E HsIFNλ4 and then fixed the following day in ice-cold methanol prior to staining for HCV NS5A antigen by indirect immunofluorescence to determine the number of cells harbouring replicating HCV RNA. Representative fluorescence microscopy and brightfield images are shown. Scale bars represent 400 μm. (TIF) [file ppat.1007307.s006.tif]

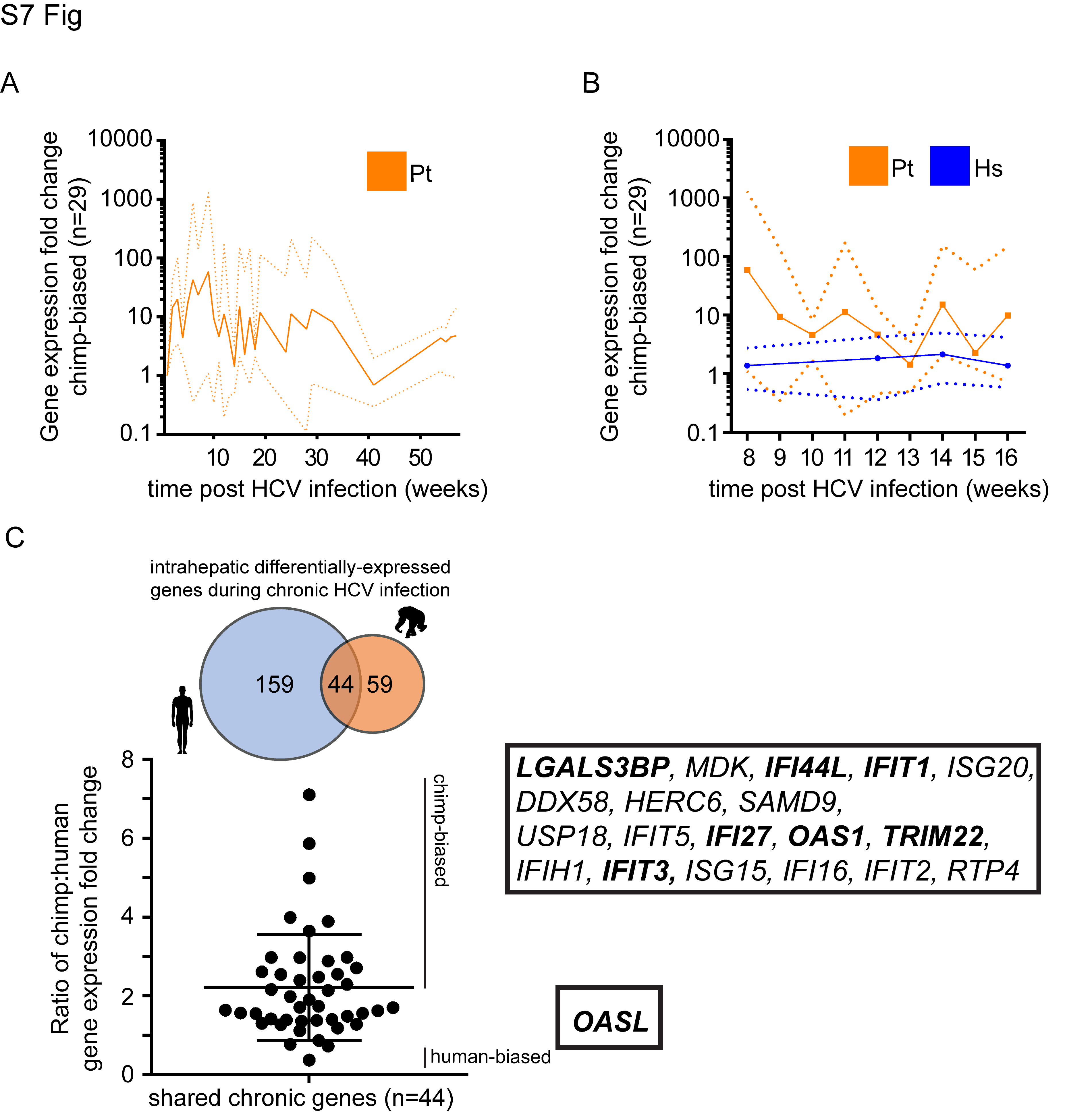

Supplement: S7 Fig — (A) Expression of ‘chimpanzee-biased’ differentially-expressed genes (n = 29) up to more than 50 weeks post infection. Chimpanzee-biased genes are shown as a combined mean (filled orange line) and range (dotted orange lines) of fold-change from all studies where any gene of the 29 genes was available over at most 1 year after initial infection. (B) Expression of differentially-expressed human and chimpanzee genes during 8 to 20 weeks post infection. Chimpanzee-biased genes are shown as a combined mean (filled orange line) and range (dotted orange lines) of fold-change from all 29 genes. The mean and range of gene expression for the human genes (blue filled and dotted lines respectively) equivalent to the chimpanzee-biased genes are shown. These data for chimpanzees are combined from different serial samples from different animals while human data is taken from a single biopsy from patients with an inferred time post infection. (C) Shared gene expression (overlapping 44 genes as shown in Venn diagram) during chronic infection in humans and chimpanzees as illustrated by a ratio of fold change in expression for humans and chimpanzees. Species biased genes (>2 fold enriched in either species) are listed to the side. All data are available in S3 Data. (TIF) [file ppat.1007307.s007.tif]

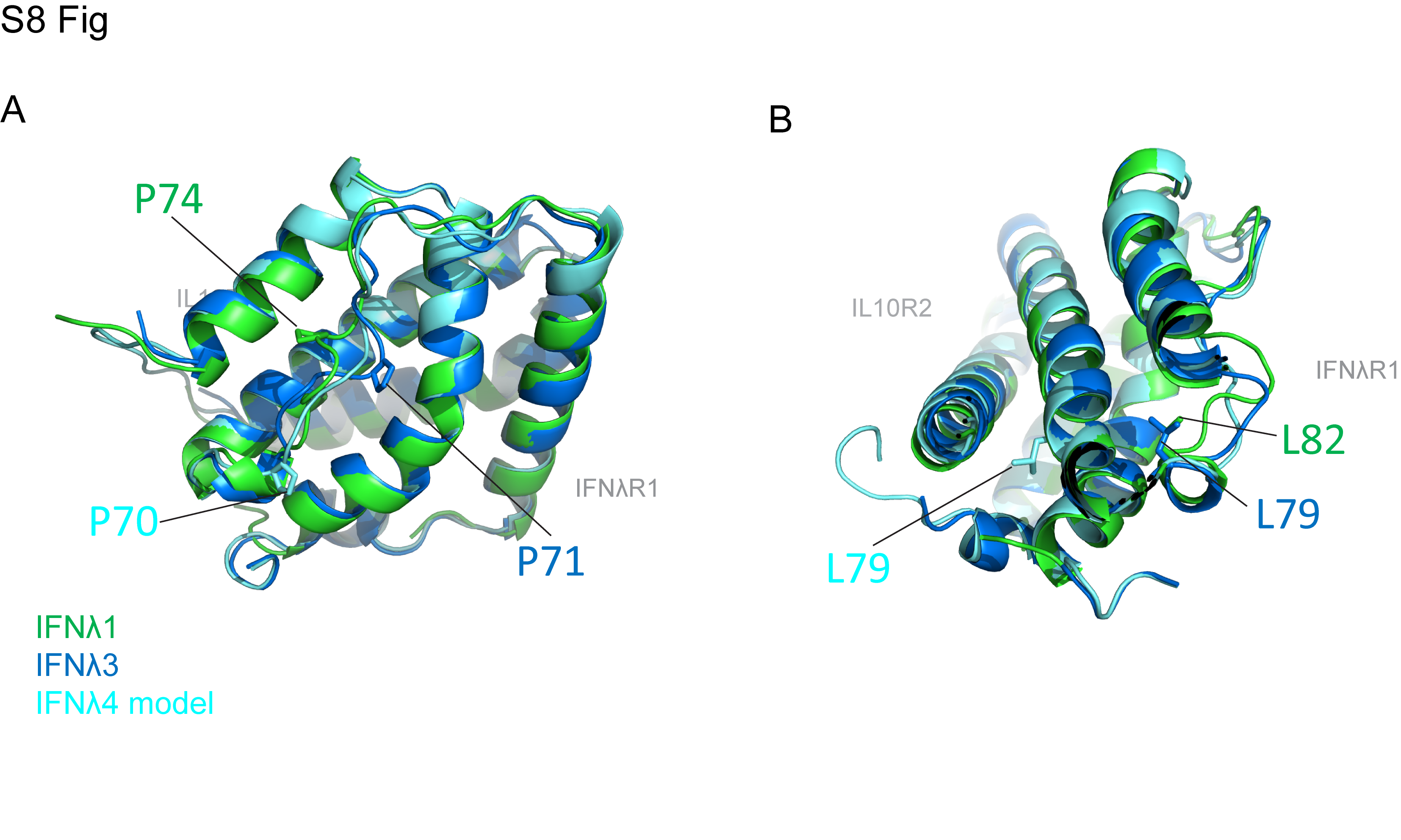

Supplement: S8 Fig — Modelled structures of HsIFNλ4 (light blue) are overlaid on the crystal structures for HsIFNλ1 (green) and HsIFNλ3 (dark blue). Panels A and B show respectively positions P70 and L79 in HsIFNλ4 and their homologous positions in HsIFNλ1 and HsIFNλ3 with reference to receptor subunit-binding interfaces (IFNλR1 and IL10R2 in grey). (A) P70 is found in a flexible proline-rich loop/helix facing the IFNλR1 receptor although no direct interactions between this region or receptor have been demonstrated. P70S might prevent required folding of the protein domain. (B) L79F is likely to disrupt packaging of the helices. Both P70 and L79 are highly conserved between IFNλ4 orthologues and paralogues (31). (TIF) [file ppat.1007307.s008.tif]

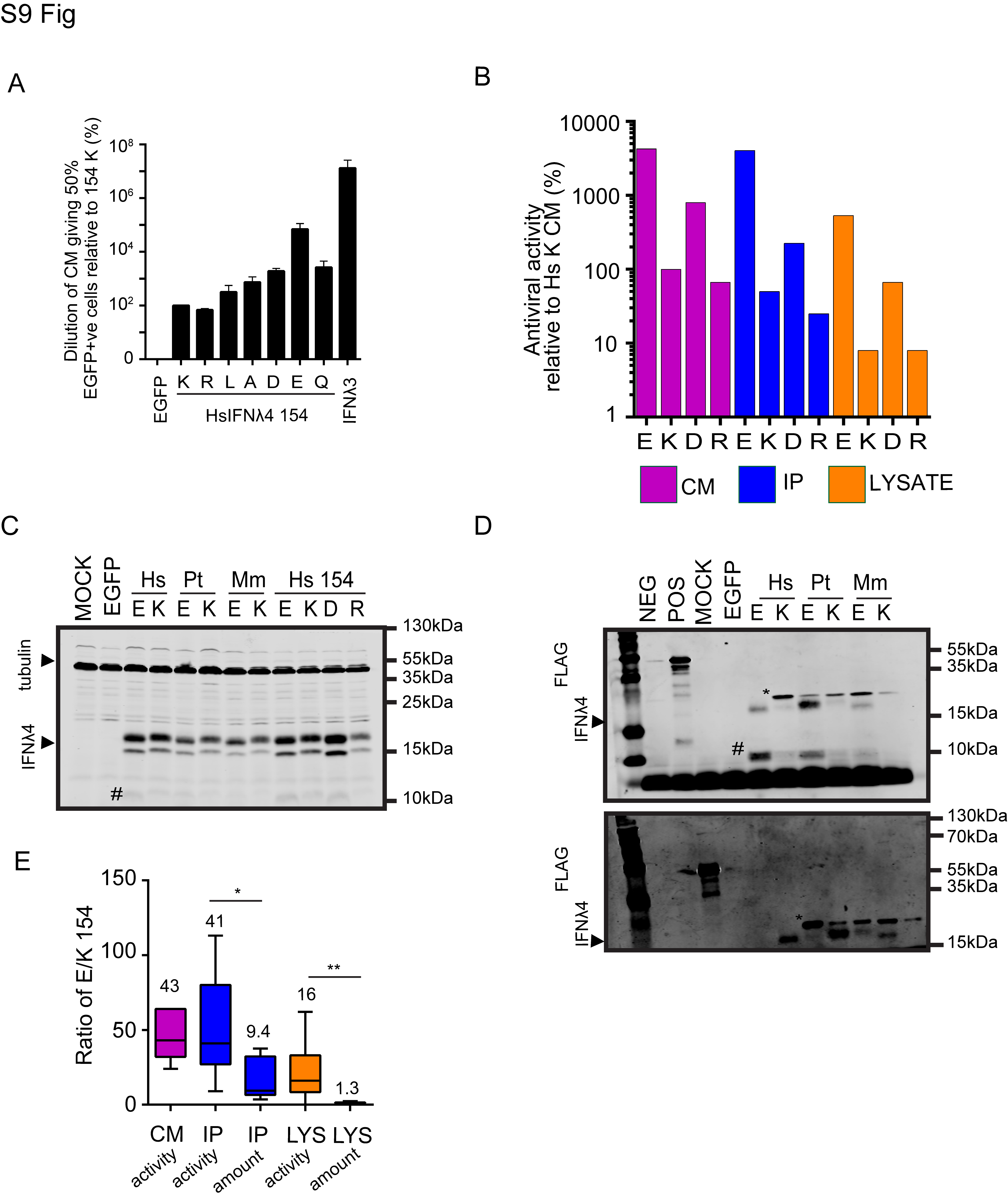

Supplement: S9 Fig — (A) Dilutions for inducing ~50% EGFP positive cells for HsIFNλ4 IFNλ4 154 mutants (R, L, A, D, E and Q) using an EGFP-ISG15 reporter cell line assay relative to CM for HsIFNλ4 K154 (%). Data show mean +/- SEM from three independent experiments performed on different days. (B) Antiviral activity of HsIFNλ4 isolated from CM (purple), intracellular lysate (blue) and immunoprecipitated CM (orange) for variants encoding E, K, R or D at position 154 in an anti-EMCV CPE assay relative to CM for wt HsIFNλ4 (K154 variant) in HepaRG cells. (C) Detection of intracellular IFNλ4 from different species as well as select mutants at position 154 (E, K, D and R) by Western blot analysis of lysates from plasmid-transfected producer HEK293T cells. The IFNλ4 variants were detected with an anti-FLAG antibody. Tubulin was used as a loading control. These samples were taken from the same experiment as in Fig 6E. A lower molecular weight product detected by the anti-FLAG antibody, which is potentially a degradation product, is highlighted with a ‘#’ (D) Alternative images from repeats of detection of extracellular IFNλ4 from different species by Western blot analysis of samples of FLAG-tag immunoprecipitated CM (1 ml) from plasmid-transfected producer HEK293T cells. A BAP-FLAG fusion protein was used an immunoprecipitation control (POS). IFNλ4 variants were detected with an anti-FLAG antibody. A lower molecular weight product detected by the anti-FLAG antibody, which is potentially a degradation product, is highlighted with a ‘#’. An upper band running near to the IFNλ4 species is shown (*) which is likely antibody fragments from the immunoprecipitation reaction. (E) Ratio of E154 to K154 for antiviral activity (all) or protein amounts (IP or LYS) of IFNλ4 found in CM, intracellular lysate and immunoprecipitated CM; where possible, data from the different species is combined. Data show median +/- minimum and maximum (n = 6–8). (TIF) [file ppat.1007307.s009.tif]

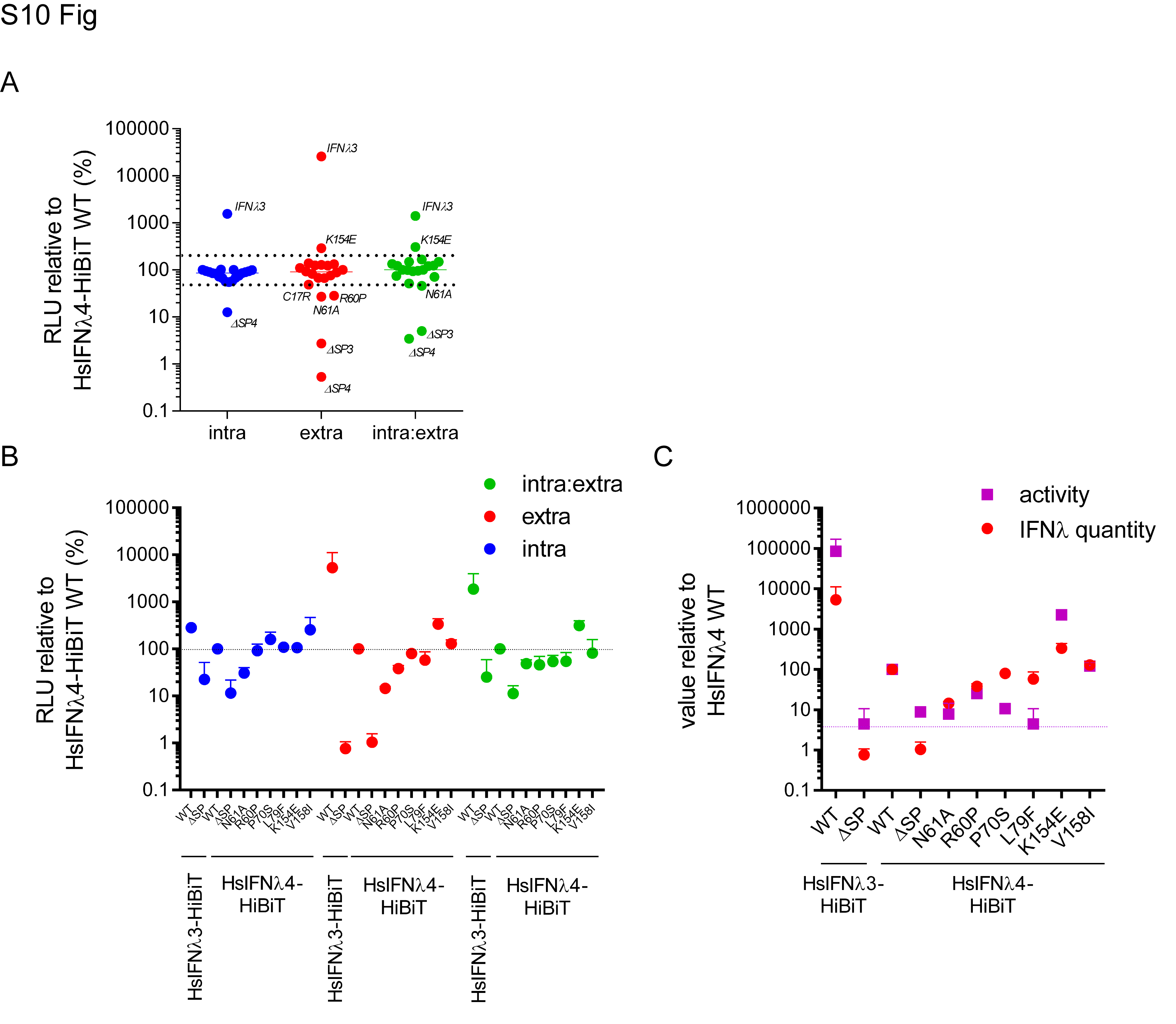

Supplement: S10 Fig — (A) Relative light unit (RLU) values, minus background measurements, for intracellular (blue) and extracellular HsIFNλs (red) as well as the ratios for these values (green) are shown relative to wt HsIFNλ4 enzyme activity for all natural HsIFNλ4 coding variants. Additional N61A (for HsIFNλ4) and ΔSP controls (ΔSP3 and ΔSP4 for HsIFNλ3 and HsIFNλ4 respectively) were included in the screen. Dashed lines indicate two-fold differences and variants giving values outside of these margins are illustrated. The data represent the mean of three independent experiments performed on different days. (B) RLU values as a validation experiment from the screen in (A) are shown following the same colour scheme for select variants of interest, including R60P, P70S, L79F, K154E and V158I. The wt value is marked time with a dashed line. (C) Paired antiviral activity for samples presented in B is shown (purple squares) relative to wt HsIFNλ4 alongside the respective amounts added (based on luciferase activity and relative to wt HsIFNλ4). A higher relative antiviral activity compared to luciferase activity indicates higher potency while the reverse is true for less potent IFNλs. The limit of detection is indicated for the antiviral activity assay (purple hashed line). The data shown in B and C represent mean and SD of biological duplicates. (TIF) [file ppat.1007307.s010.tif]

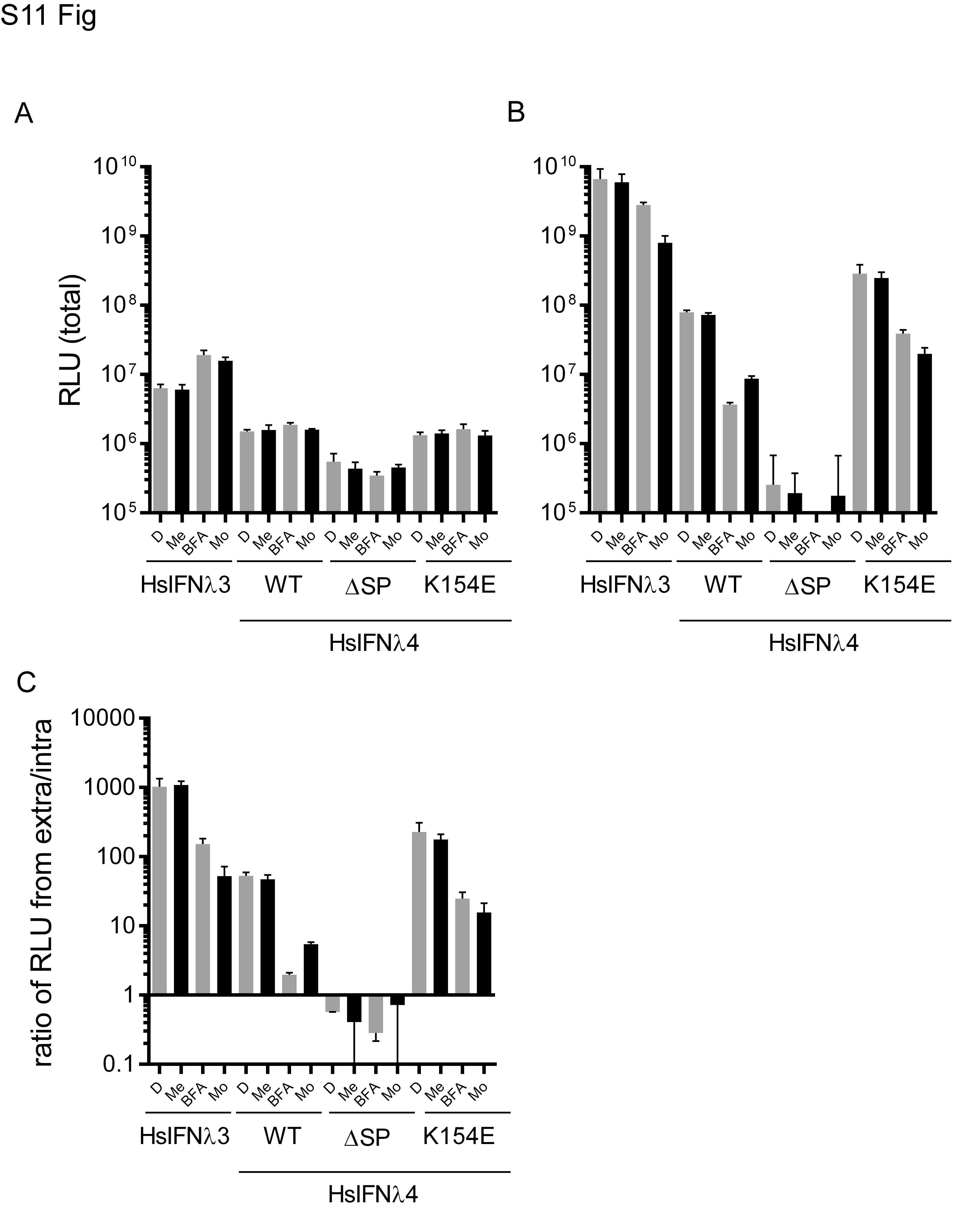

Supplement: S11 Fig — Raw total RLU values minus the mock treated from within (A) and outside (B) the cell are shown following treatment of transfected cells with two inhibitors of the secretion pathway, brefeldin A (BFA) and monensin (Mo); appropriate negative controls using DMSO and methanol respectively for each compound are shown. Both inhibitors were used at a final concentration of 5 μg/ml. HEK-293T cells transfected with plasmids were treated with inhibitors and controls at ~18 hpt and incubated for a further 24 hrs in the presence of the drug before luciferase activity were measured. In this experiment, wt HsIFNλ3 and wt HsIFNλ4 alongside the K154E variant and controls lacking the signal peptide sequence were used. (C) The extent of secretion for HsIFNλs are shown as ratios of intracellular and extracellular luciferase values from the experiment in A and B. Histograms show mean and SD from wells in triplicate. (TIF) [file ppat.1007307.s011.tif]

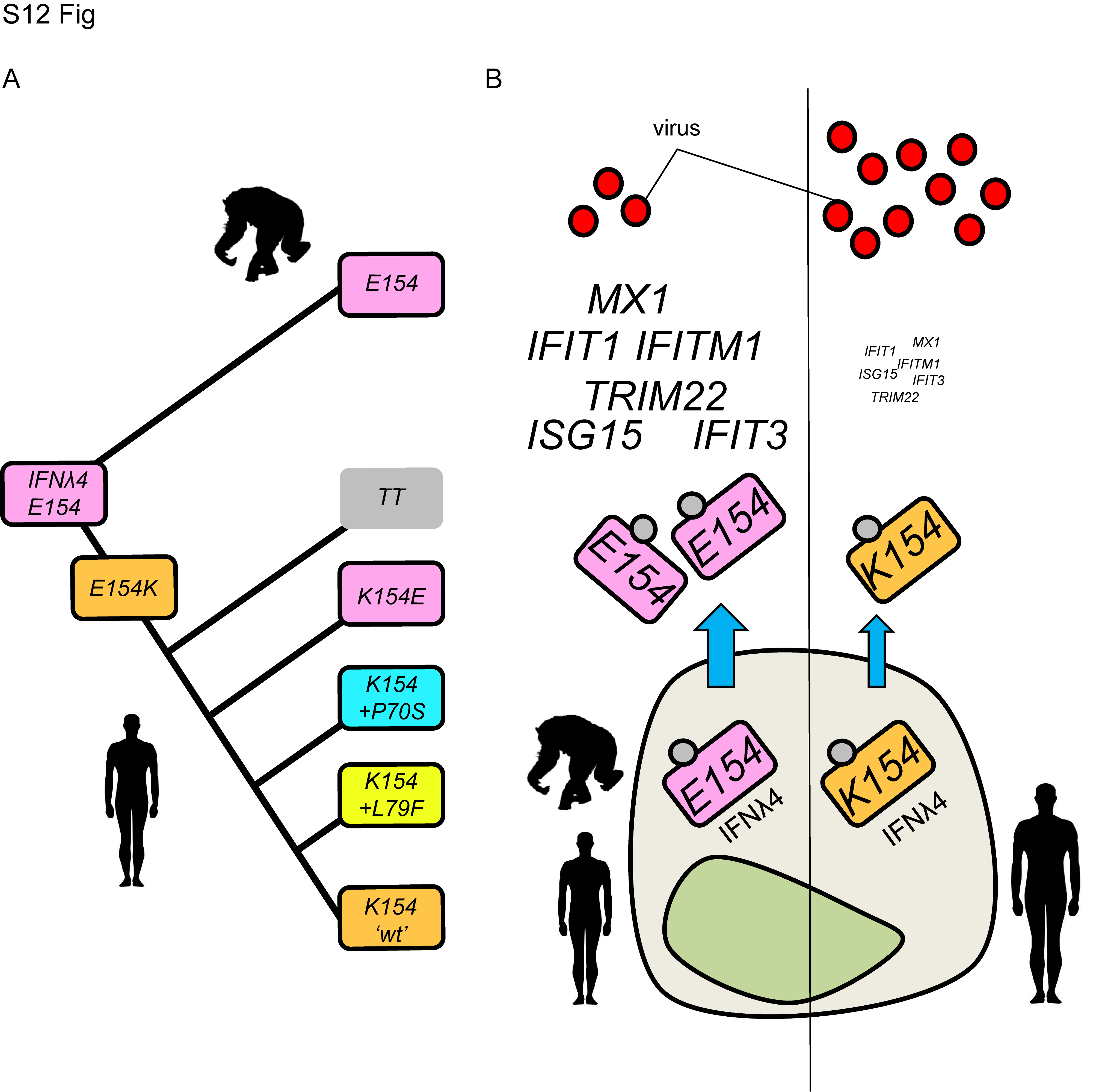

Supplement: S12 Fig — (A) Inferred evolution of position 154 in humans and chimpanzees. The last common ancestor of humans and chimpanzees encoded the highly-conserved glutamic acid (E) at position 154 (purple). E154 was retained in chimpanzees but sequentially modified in the genus Homo, which includes humans and Neanderthals. Homo IFNλ4 was first modified by substitution of E154 to lysine (K) (orange) and subsequent emergence of the frameshift TT allele (grey) or by the introduction of other substitutions that reduce activity (P70S [blue], L79F [yellow]). The TT allele also encodes the codon for K154. E154 (pink) re-emerged in Pygmies.IFNλ4 in humans with only the E154K change remains in the population and is considered wild-type ('wt'). (B) Impact of E and K encoded at position 154 in IFNλ4 on antiviral activity. Both IFNλ4 E154 (purple) and K154 (orange) are produced and glycosylated (grey circle) to similar levels inside the cell but IFNλ4 E154 is secreted more efficiently compared to IFNλ4 K154 (highlighted by blue arrows) and has enhanced potency. Subsequently, IFNλ4 E154 induces more robust interferon stimulated gene (ISG) expression (for example: ISG15, IFIT1, MX1) in target cells, leading to greater antiviral activity. (TIF) [file ppat.1007307.s012.tif]

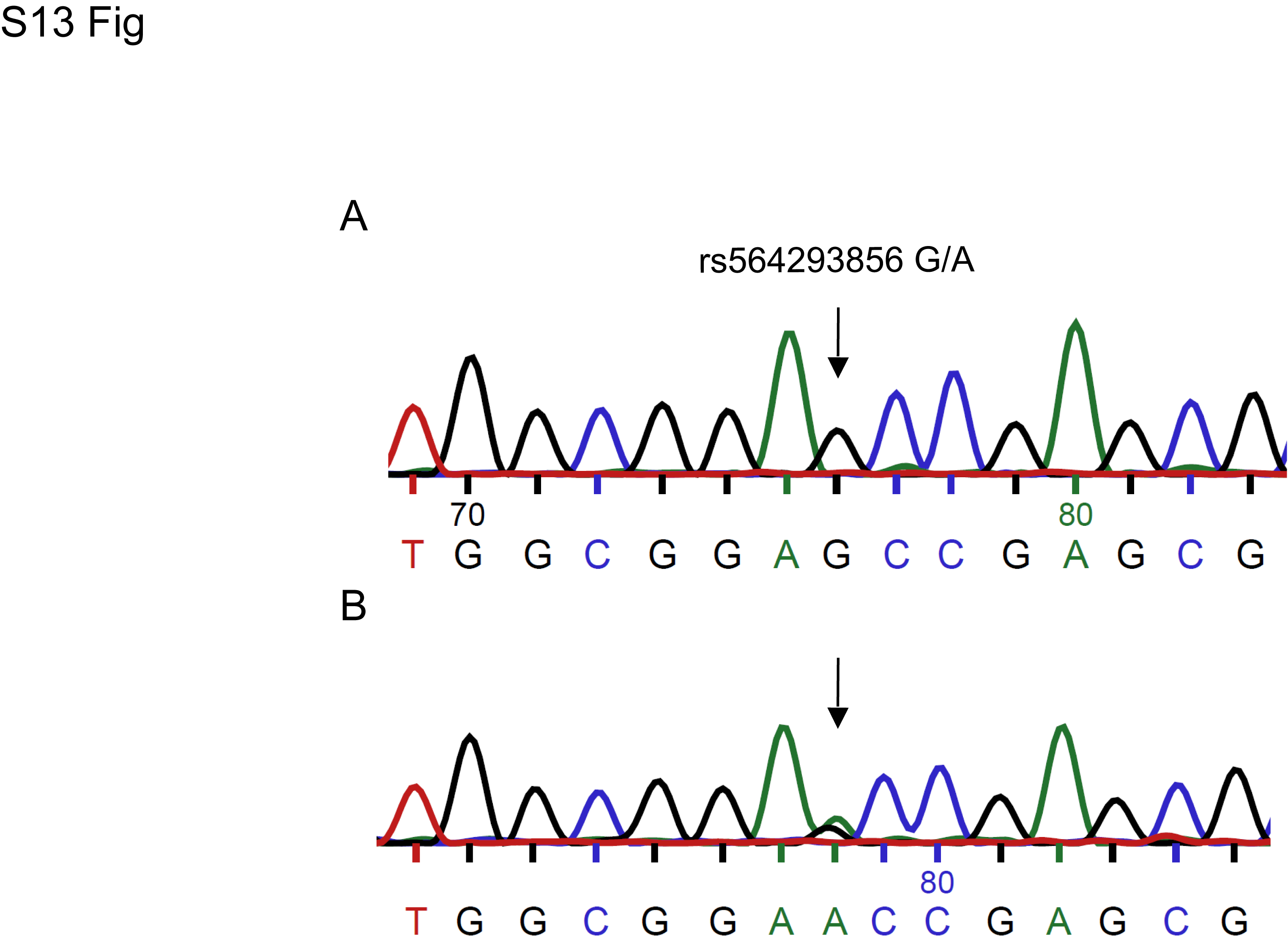

Supplement: S13 Fig — (A) Chromatogram from an amplicon showing the region surrounding the rs564293856 variant for the American proband (NA19658) with no evidence of a G>A allele (highlighted with the vertical black arrow). (B) Chromatogram from an amplicon showing the region surrounding the rs564293856 variant for the African proband (HG03095) showing evidence for both G and A nucleotides at rs564293856 (highlighted with the vertical black arrow). This individual was heterozygous as predicted. (TIF) [file ppat.1007307.s013.tif]
